# Biomedical Applications of Carbon-Based Nanomaterials: Exploring Recent Advances in Therapeutics, Diagnostics, and Tissue Engineering

**DOI:** 10.34172/apb.025.44083

**Published:** 2025-05-31

**Authors:** Afsona Parveen, Arnab Chatterjee, Prithviraj Karak

**Affiliations:** ^1^Department of Bachelor in Medical Laboratory Technology, Durgapur Institute of Paramedical Science, West Bengal, India; ^2^Department of Nutrition, Asansol Girls’ College, West Bengal- 713304, India; ^3^Department of Physiology, Bankura Christian College, West Bengal-722101, India

**Keywords:** Carbon-based nanoparticles, Biomedical applications Graphene, Fullerene, Carbon nanotubes

## Abstract

Carbon-based nanoparticles possess distinctive chemical, physical, and biological characteristics that render them suitable for biomedical uses. This paper reviews recent advancements in carbon-based nanomaterial (CBs) synthesis methods, emphasizing the importance of careful modification for biomedical uses, particularly in the passivation of drugs and chemicals on their surfaces. This review article examines information from 2021-2024 regarding carbon-based nanoparticles and the biomedical uses of graphene, fullerene, carbon nanotubes, nano horns, nanodiamonds, quantum dots, and graphene oxide. Initially, a total of 5,612 relevant data points from various databases such as PubMed, ScienceDirect, and Web of Science were analyzed. After eliminating duplicates, nearly 3,905 data points were found to meet the inclusion criteria for this study, with the latest research indicating that 1,791 (45.8%) of these databases pertained to graphene. Carbon nanotubes accounted for approximately 928 (25.14%) databases, while graphene oxide represented around 837 (21.43%) databases, placing them in second and third positions, respectively. Nanohorns and fullerene were found in very minor quantities, specifically 34 (0.87%) and 06 (0.15%) in the database. CBNs, have the capacity to revolutionize biological medicine by improving regenerative treatments, personalized healthcare, and therapeutic outcomes. They are utilized in scaffolding, drug delivery, tissue engineering, bioimaging, and additional fields. Nonetheless, successful integration necessitates tackling scale and regulatory limitations.

## Introduction

**Figure 1:**
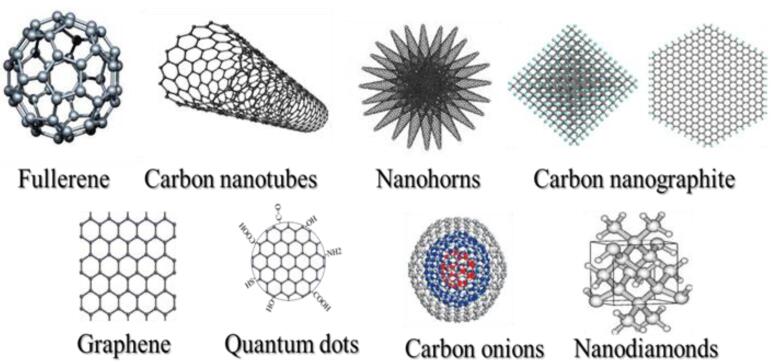
Structures of various carbon based nonmaterial (CNMs)

 In light of their special physicochemical qualities, carbon-based nanomaterials (CBNs) have become attractive options for a variety of biological applications.^1^ The potential applications of these materials—which include graphene, fullerenes, nano-diamonds, nanohorns, nanotubes, quantum dots, carbon onions, and carbon nanotubes (CNTs) (Figure 1) -in tissue engineering, drug delivery, biosensing, imaging, and cancer treatment have attracted a lot of interest.^2^ For targeted drug delivery and therapeutic applications, their large surface area, superior biocompatibility, and changeable surface chemistry make them perfect.^3^ Furthermore, because of their extraordinary optical qualities, CBNs have demonstrated considerable promise in diagnostic imaging methods, including photoacoustic imaging and fluorescence imaging.^4^ These Nanomaterials have enormous potential to transform biomedicine and enhance patient outcomes with more study and development.^5^ The application of CBNs to improve the effectiveness of cancer treatment has been the subject of recent research.^6^ These Nanomaterials’ special qualities allow them to act as carriers for chemotherapeutic medications, minimizing systemic side effects and enabling targeted delivery to tumor sites. Furthermore, Graphene and CNTs have demonstrated promise in photothermal therapy, which uses the materials’ ability to absorb light to specifically kill cancer cells.^7^ Furthermore, the potential of CBNs in biosensing and diagnostic applications has been a subject of intense research.^8^ Their ability to interact with biological molecules and cells has led to developments in biosensors for detecting biomarkers associated with various diseases, including cancer, cardiovascular disorders, and infectious diseases.^9^ Furthermore, CBNs may be functionalized with targeting ligands and imaging agents such as peptides (RGD peptides), antibodies (anti-HER2), and aptamers (DNA aptamers), due to their changeable surface chemistry, which makes them useful for targeted treatment and molecular imaging.^10^ To fully realize the promise of CBNs in biomedical applications, multidisciplinary teams in the field of nanomedicine must continue their research and collaborate.^11^ These Nanomaterials have the potential to greatly care and customized medicine via progress in their synthesis, characterization, and comprehension of their interactions within biological systems.^12^

## Synthesis of carbon-based nanomaterials

**Figure 2:**
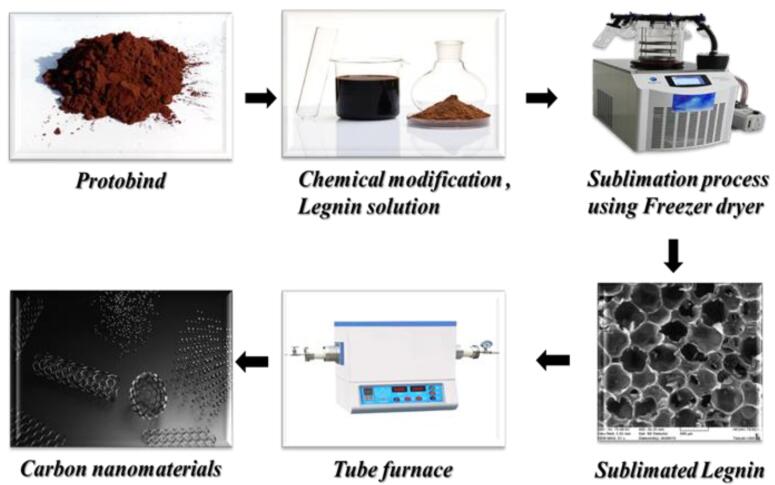
Synthesis procedure of carbon-based nanomaterials

 At present, emerging technology can easily regulate environmental pollution, energy generation, agricultural development, and food production, resolving global concerns and paving the way for a bright future for humanity.^13^ In response to this, scientists have discovered a wide range of methods for synthesizing CBNs, which they then employ in emerging technologies.^14^ One of those CBNs called “fullerene” is employed extensively in most scientificfields, although scientists are still unsure of how it is made.^15^ Though popular methods for this goal include the vaporization of graphite electrodes, hydrocarbon pyrolysis, and laser ablation.^16^ Fullerenes are separated from soot using solvents, and the product is processed using liquid or column chromatography.^17^ Electrochemical or solution ozonolysis processes are now being introduced by emerging technologies for synthesis purposes.^18^ (Figure 2).

 “Carbon onion” is another CBNs that is created using a variety of methods, including chemical vapor deposition (CVD), electron irradiation, and nanodiamond transformation.^19^ Acetylene, boron trichloride, and ammonia constitute some of the precursors that are broken down by CVD, which uses hydrogen ions as a carrier molecule.^20^ An alternative method of creating CNOs is to build a graphitic outer shell by heating a diamond core to 1700 °C in a vacuum.^21^ The diamond phase transforms into a facetted graphitic structure as the temperature rises.^22^ The type of precursor and the conditions of synthesis have a significant impact on the structure of carbon onions; all carbon onions have a multi-shell architecture similar to that of fullerenes.^23^

 Another type of CBN known as “nanodiamond,” which is a classification of diamond, is created by top-downly breaking up bulk diamonds.^24^ The bulk diamonds and these nanodiamonds have similar mechanical, optical, thermal, electrical, and biocompatibility properties.^25^ Nevertheless, a number of studies have recently used CVD techniques to create nanodiamonds.^26^ CBNs, known as carbon quantum dots (CQDs), may be synthesized using many procedures, including electrochemical, top-down, arc discharge, laser ablation, combustion, and microwave pyrolysis.^26,27^ Additionally, those nanomaterials are cut utilizing top-down methods, and electrochemical exfoliation and arc discharge are used to create cheap carbon electrodes.^26,27^ The discovery of CNTs, another kind of CBN, in fullerenes led to the development of several methods for producing single and multiwalled CNTs.^28^ Other methods include laser ablation, which uses Nd: YAG and CO_2_ lasers to melt graphite targets, and CVD, which breaks down hydrocarbon precursors in the presence of a metallic catalyst. Graphene is a carbon-based material that is synthesized using a single sheet structure and two distinct ways called bottom-up and top-down approaches.^29^ Among the methods employed in the bottom-up process include CVD, plasma-enhanced CVD, and epitaxial growth on silicon carbide (SiC). In top-down synthesis, superior graphite crystals are separated into graphene sheets by mechanical or chemical means.^30^

## Functionalization of carbon-based Nanomaterials

 Researchers can easily functionalize CBNs with the help of modern, advanced technologies by improving their properties and making them more useful in many fields, including the biomedical field.^31^ This will help people and the next generation of health care facilities.^32^ As mentioned before, there are already a variety of CBNs available, fullerenes being one of them. Typically, there are three methods to functionalize fullerenes: grafting hydroxyl groups, covalent functionalization, and surface functionalization.^32^ Surface functionalization causes fullerene to become soluble in organic solvents and water.^32,33^ There are, however, two more types of fullerene covalent functionalization: complexation with a solubilizing agent and covalent fictionalization.^33^ Strong acids and high temperatures can be used to graft oxygen-based functional groups—mainly hydroxyl groups—onto fullerene surfaces. Due to their biocompatibility and resistance to cell differentiation, development, and proliferation, another carbon-based group of diamonds, these minerals is frequently utilized for a variety of purposes, such as sensing purposes for nanostructures, mass spectrometry, chromatography, tribology, electroanalysis, lubrication, creating fluorescent tags for analyzing biological processes, locating microscopic substances, and energy storage.^15,34^

 CNTs, another form of nanomaterial based on carbon, have undergone several functionalization’s. CNTs and functional groups (Diels-Alder cycloaddition, carbene and nitrene addition, chlorination, bromination, azomethine ylides, and hydrogenation) form covalent linkages to give acidic sites for attachments during the covalent fictionalization process.^34^ In spite of this, covalent functionalization has the potential to break the aromatic ring of CNTs and modify their electrical and mechanical properties.^35^

 While supramolecular complexation, adsorption, and biomolecules preserve the non-covalent functionalization of CNTs, their structural and electrical properties are still preserved. In order to ascertain its properties and its applications in electronics, biomaterials, sensing, energy, and the environment, graphene—the most demanding CBN—must undergo chemical fictionalization.^34,36^ Zero-gap graphene transforms into semiconductors for use in biomaterials and electrical applications; it may also be used as an electrode material to boost the efficiency of electrocatalysis.^36^

 Graphene oxide (GO) undergoes chemical modifications by grafting, radical additions, electrophilic substitution, and cycloaddition reactions in the case of covalent fictionalization.^37^ Organic compounds, quantum dots, and polymers are used to functionalize GO.^34,38^ When non-covalent functionalization occurs, interactions between counter molecules preserve the graphene’s linear structure. Because of its properties, including its high strength, flexibility, and reversible tensile elastic strain, graphene is a useful material for sensors.^38^

## Bioavailability and toxicity of carbon-based Nanomaterials

 Future generations might benefit greatly from the use of CBNs in industries such as agriculture, food production, food safety, nanomedicine, pharmacy, the drug industry, and the biomedical area.^39^ Nevertheless, there’s also evidence that CBNs have harmful effects, which may be controversial for now a day.^40^ Researchers found that overexposure to CBNs create toxicity in the environment as well as to human health.^40^

**Figure 3:**
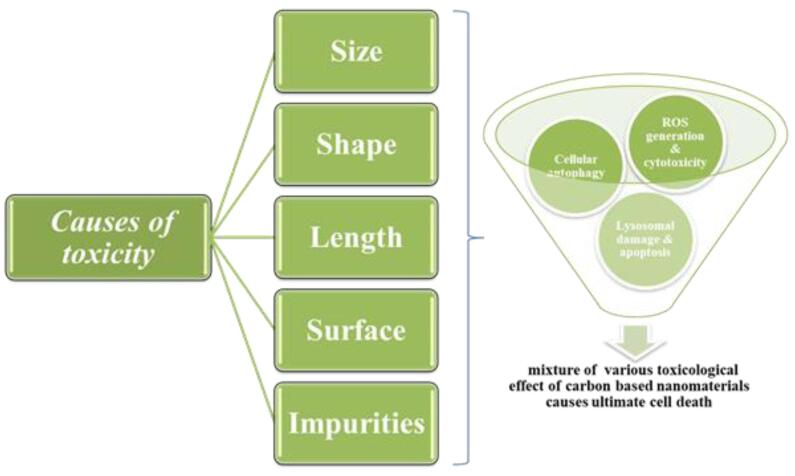
Cause and effect of toxicity of carbon-based nanomaterials

 Experts have discovered that the physical characteristics of CBNs, such as their length, size, shape, surface functionalization, and impurities influence their toxicity and bioavailability^41^ (Figure 3). Research has shown that longer CBNs are more cytotoxic than shorter ones, decreasing cell viability and increasing ROS production. On the other hand, the toxicity of CBNs is inversely related to their size.^42^ Differently shaped carbon nanoparticles have different dangerous properties.^43^ While both SWCNTs and MWCNTs exhibit significant harmful effects at lower doses, SWCNTs are much more cytotoxic than MWCNTs at the same concentration. Since GOs differ from SWCNTs in their physical characteristics, they pose a greater risk.^43^

 The toxicity of carbon nanomaterials may be more complex than their shape; for example, longer MWCNTs are more lethal than spherical ones.^44^ Carbon nanomaterial toxicity is arranged in a broad sequence.^45^ When nanoparticles are surface functionalized, it has a deleterious effect on immune-mediated cells such as dendritic cells, macrophages, and lymphocytes.^45^ The production of ROS, cellular autophagy, lysosomal damage, pyroptosis, apoptosis connected to mitochondrial pathways and scavenger receptors, and cellular necrosis are among the underlying mechanisms of CBNs’ cytotoxicity (Figure 3).^46^

 Fullerene, a CBN primarily found in aquatic invertebrate animals, along with graphite, acts on a variety of target cells in the human body, including mesenchymal cells, especially dermal fibroblasts, HepG2, neuronal human astrocytes, alveolar macrophages, and human monocyte-derived macrophages.^47^ This cytotoxicity results in the release of lactate dehydrogenase, disruption of cellular membranes and lipid peroxidation, decreased cell viability, and apoptosis/necrosis.^48^ Among carbon-based nanotubes, it has been discovered that multiwalled carbon nanotubes (MWCNTs) cause cellular toxicity, whereas single-walled carbon nanotubes (SWCNTs) exhibit cytotoxicity in a concentration-dependent way.^49^ Research has demonstrated that MWCNTs cause a considerable reduction in cellular phagocytosis as well as apoptosis.^50^

## Methodology

###  Search approach and data selection specifications

 Several credible scientific search engines are employed including Pub-Med was the primary database for the searches, however we also searched like MEDLINE, NDSL (National Digital Science Library) and was limited to English-language papers published in peer-reviewed journals or conference proceedings between 2021 and 2024 to gather information and conduct searches for this review article. Recommendations for meta-analyses are also employed, along with the suggested reporting items for evaluation. These websites are used to gather information for this study and references from pertinent publications by utilizing the search criteria “carbon-based nanoparticles,” “biomedical applications of graphene,” “biomedical application of fullerene,” “biomedical application of carbon nanotubes,” “biomedical application of nanohorns,” “biomedical application of nanodiamonds,” “biomedical application of quantum dots,” and “biomedical application of graphene oxide.” Data screening was done from the list of chosen studies.

###  Database search protocol and keywords

 The study article’s search was conducted in a repeating manner. The article’s introduction contains a number of important phrases, including “Carbon nano horns,” “special chemicals,” “carbon dots,” “carbon,” “earth,” “treatment,” “nanomaterials,” “graphene,” and “biomedical application.” During future versions, the article’s title was carefully examined to identify more optimal keywords, such as “carbon,” “nanomaterials,” “biomedical applications,” etc.

###  Data synthesis, extraction, and inclusion/exclusion standards

 The information was retrieved from a variety of scientific articles that had been published in “English” in respectable publications and scientific search engines during 2021 and 2024, which included 1. Studies that focused on the biomedical applications of CBNs, including therapeutics, diagnostics, and imaging. 2. Studies that reported on the use of CBNs for biomedical applications, including in vitro, in vivo, and clinical trials. 3. Studies that provided sufficient detail on the synthesis, characterization, and application of CBNs.

 To fully meet the objectives, the main conclusions of the information extraction, patterns, and trends are further examined. The exclusion criteria were, 1. Studies that did not focus on the biomedical applications of CBNs. 2. Studies that did not report on the use of CBNs for biomedical applications. 3. Studies that were published outside the specified time frame or were not published in English. 4. Studies that did not provide sufficient detail on the synthesis, characterization, and application of CBNs.

 The data presented here is made clearer and more impactful by the use of charts, graphs, and other visual components. In order to mitigate the potential for bias, the reviewers reviewed this article. Reviewers utilized methods like EndNote or manual screening to remove duplicate articles from multiple databases, ensuring the analysis was based on unique studies, enhancing the accuracy and reliability of the findings.

 The study outlined steps to manage duplicate data points, including exclusion of duplicate studies, de-duplication of extracted data points, and data aggregation. Sensitivity analyses on some overlapping datasets also performed to assess the robustness of the findings and established a dataset hierarchy prioritizing comprehensive and up-to-date ones to minimize the impact of overlapping data.

## Results

 Around 5612 relevant data were found for examination of this article. From which 1707 data are found to be duplicate and removed. The publications were screened and found 3905 data points using their title and abstracts. From which around 817 data were found from PubMed database, 2403 data were found from ScienceDirect database, and 685 From Web of science database (Table 1). Among them majority were removed for full filling exclusion criteria and only few were found which meet inclusion criteria.

**Table 1:** Number of data sets of carbon-based nanomaterials from various scientific search engines.

**SL.** **No.**	**Carbon-based nanomaterials**	**Scientific search engines**		
		**PubMed **	**ScienceDirect**	**Web of science**
01	Graphene	404	787	600
02	Fullerene	05	00	01
03	Carbon nanotubes	172	745	11
04	Nanohorns	03	21	10
05	Nano diamonds	27	78	33
06	Quantum dots	140	06	25
07	Graphene oxide	66	766	05

**Figure 4:**
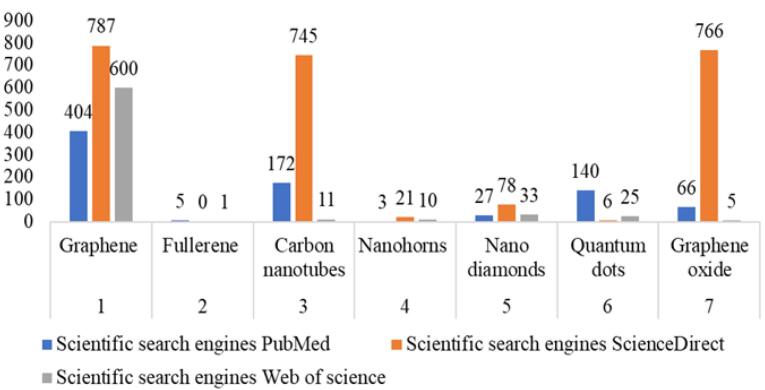
Comparative study outcomes of different carbon-based nanomaterials and their biomedical applications from various search engines

 Bar graphs showing comparative study outcomes of different CBNs and their biomedical applications from various search engines (Figure 4). Graphene-based nanomaterials were the most commonly used CBNs, accounting for approximately 45.8% of the studies reviewed. CNTs and graphene oxide were the second and third most frequently used CBNs, representing around 25.14% and 21.43% of the studies. Fullerenes and nanohorns were less commonly used, but still showed promise in specific biomedical applications. In the PubMed database, graphene has the most studies in recent years, followed by quantum dots and CNTs. PubMed found very little data regarding fullerene and nanohorns. According to the ScienceDirect search engine, graphene likewise exhibits very demanding research, with graphene oxide and CNTs coming in second and third. The ScienceDirect database contained no additional information about fullerene’s use in biomedicine. Graphene also has a large number of search results in the Web of Science database, while other carbon nanomaterials have the fewest studies in recent years.

## Discussion

###  Biomedical applications of carbon-based Nanomaterials

**Figure 5:**
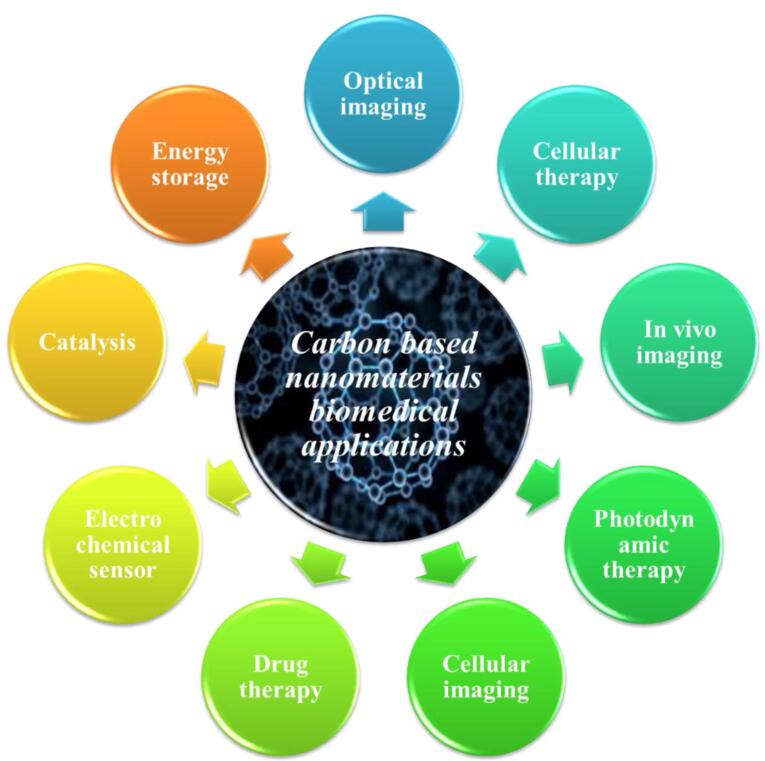
Biomedical application of carbon-based nanomaterials

 In addition to their special qualities, CBNs specially CNTs and graphene are materials with different properties and advantages for therapeutic applications have drawn a lot of interest in biological applications (Figure 5).^51^ These materials have exceptional mechanical strength, great electrical and thermal conductivity, and a vast surface area.^52^ Examples of these materials are graphene, fullerene, and CNTs.^53^ CNTs have a high aspect ratio, excellent mechanical strength, and can be functionalized for biocompatibility and specificity. Graphene has a high surface area, excellent electrical conductivity, and thermal properties. However, they have potential toxicity, difficulty in uniform dispersion, and concerns about long-term biocompatibility. Graphene has potential toxicity due to high reactivity and oxidative stress, and challenges in scaling production. Both materials have potential applications in drug delivery, tissue engineering, and biosensing. The choice between them depends on the specific application and addressing their limitations. CBNs have demonstrated potential in improving the therapeutic effectiveness of medication delivery.^51,53^

 Nonetheless CBNs offer potential for personalized medicine by targeting specific genetic mutations or profiles. By using targeted ligands that are aware of particular biomarkers or receptors, these nanoparticles can be functionalized. Applications include gene therapy, cancer treatment, and precision medicine. Improved therapeutic effectiveness, fewer adverse effects, and individualized medication are among the advantages. By developing customized therapies that cater to the particular requirements and genetic characteristics of each patient, researchers can improve the quality of healthcare overall.

 Effective medication loading and tailored distribution to certain tissues or cells are made possible by the increased surface area.^54^ Additionally, they are appropriate carriers for a variety of pharmacological substances due to their biocompatibility and capacity to cross cell membranes. Additionally, carbon-based nanoparticles are essential to biosensing technology.^55^

 Their high electrical conductivity and large surface-to-volume ratio enable the detection of biomolecules with exceptional sensitivity.^56^ This capability has profound implications for diagnostic tools, environmental monitoring, and medical devices. In the realm of medical imaging, CBNs have emerged as contrast agents due to their unique optical and magnetic properties.^57^ These materials hold promise for improved resolution and specificity in imaging modalities, including magnetic resonance imaging and fluorescence imaging.^58^

 Moreover, in tissue engineering, CBNs contribute to the development of scaffolds with enhanced mechanical properties and bioactivity, fostering tissue regeneration and repair.^59^ The multifaceted applications of CBNs in biomedicine underscore their potential to revolutionize healthcare technologies and contribute to advancements in therapeutic interventions and diagnostics.^60^

 Biomolecules may be detected with remarkable sensitivity thanks to their enormous surface-to-volume ratio and strong electrical conductivity.^61^ This feature has significant effects on medical equipment, environmental monitoring systems, and diagnostic instruments.^62^ Because of their special optical and magnetic characteristics, CBNs have become popular contrast agents in the field of medical imaging.^62^ Magnetic resonance imaging and fluorescence imaging are two imaging modalities where these materials show potential for better resolution and specificity.^63^

 With different thresholds according on the kind, cell line, and exposure time, CNMs can cause cytotoxicity, genotoxicity, inflammation, and oxidative stress. They may also cause oxidative stress and inflammatory reactions, which can harm tissue. In order to create safer and more efficient nanomaterials for biomedical applications, it can be helpful to comprehend their toxicity and biological implications.

 Furthermore, CBNs support tissue regeneration and repair in tissue engineering by helping to create scaffolds with improved mechanical and biological qualities.^64^ The potential of CBNs to transform healthcare technology and enhance therapeutic treatments and diagnostics is highlighted by their many uses in biomedicine.^65^

###  Drug delivery

 Nanomaterials based on carbon have demonstrated enormous promise for transforming medication delivery methods. Their special qualities make them ideal for improving the medicines’ targeted delivery and effectiveness in a range of biological applications.^66^ Pharmaceutical substances may be efficiently loaded onto CBNs due to their large surface area.^67^ This characteristic makes it possible to encapsulate and provide a wide variety of medications, including proteins, nucleic acids, and tiny compounds.^68^ Furthermore, the broad surface area makes it easier for targeting ligands to adhere, allowing for targeted distribution to sick tissues or cells with the least amount of systemic adverse effects.^69^ CBNs have the potential to improve the effectiveness of therapies by providing accurate control over pharmacokinetics and drug release kinetics.^70^

 With the prolonged and targeted drug administration provided by this controlled release mechanism, treatment results are enhanced, dosage frequency is decreased, and side effects are minimized.^71^ The biocompatibility and cell membrane penetration of carbon-based nanoparticles constitute two further noteworthy advantages.^72^ These characteristics improve the administration of medicines, even those with low solubility or restricted cellular absorption, intracellularly by facilitating the passage of pharmaceuticals through biological barriers and into target cells.^73^ Moreover, combination treatment can target complicated disorders because of the adaptability of CBNs, which enable the co-delivery of many medications or therapeutic agents.^74^ The capacity to create customized medication delivery systems based on genetic and molecular profiles to meet the demands of individual patients offers great promise for personalized medicine such as targeted cancer theraphy.^75^

 Therefore, the use of CBNs in drug delivery is a novel strategy for improving therapeutic treatments.^76^ CBNs have shown promise in treating diseases like cancer. CNTs can target cancer cells, deliver chemotherapeutic agents, and reduce side effects. Graphene oxide nanoparticles can target tumors, use photothermal therapy, and deliver therapeutics to specific cells or tissues. Carbon nanodots can be conjugated with targeting molecules and used for imaging and therapy. Precision medicine and individualized drug delivery tactics will rely heavily on these nanomaterials because of their capacity to increase therapeutic effectiveness, facilitate targeted distribution, and optimize drug loading.^77^ Researchers can develop targeted drug delivery systems to improve treatment outcomes and reduce side effects.

###  Biosensing

 CBNs have attracted a lot of attention in the field of biosensing because of their many uses in the highly sensitive and selective biomolecule detection and quantification processes.^78^ Their special qualities, such as their vast surface area, high electrical conductivity, and biocompatibility, have made them useful parts of biosensing technologies.^79^ The creation of electrochemical biosensors is one of the main uses of CBNs in biosensing.^80^ These biosensors identify and measure biological analytes by taking advantage of the electrical characteristics of carbon nanomaterials like graphene and CNTs.^80^ These nanomaterials’ high electrical conductivity facilitates quick electron transfer, which makes it possible to detect biomolecules—such as proteins, DNA, and tiny molecules—selectively and sensitively.^81^

 Moreover, CBNs are essential to optical biosensing systems because they use their special optical characteristics to identify biomolecules.^82^ For instance, graphene’s remarkable optical transparency makes it possible to create biosensors without labels.^83^ Furthermore, carbon nanomaterials’ surface plasmon resonance capabilities allow for the real-time observation of biomolecular interactions, which makes them useful instruments for fields like drug development and medical diagnostics.^84^ The development of sophisticated biomedical diagnostic instruments is greatly encouraged by the use of CBNs in biosensing.^85^ Due to their high surface-to-volume ratio and biocompatibility, they may be able to help with early illness diagnosis and monitoring by enabling the sensitive detection of disease biomarkers.^86^

 Furthermore, by offering quick and precise diagnostic capabilities, the use of these nanomaterials in point-of-care devices has the potential to completely transform the healthcare industry, especially in environments with limited resources.^87^ CBNs are used in biosensing technologies for environmental monitoring, in addition to biomedical applications.^88^ They are useful for monitoring the quality of water and air, agricultural pollutants, and the general health of the environment because of their sensitivity to changes in the environment and their capacity to identify particular pollutants or biomolecules.^89^ Biosensing innovation has enormous promise as long as research on CBNs continues to progress.^90^ With far-reaching implications for healthcare, environmental sustainability, and biotechnology, the outlook for using CBNs in biosensing appears promising.^91^ Examples of these include the development of wearable biosensors for continuous health monitoring and the integration of nanomaterial-based biosensing platforms in smart medical devices.^92^

###  Sensing implants

 An intriguing new area in biomedical engineering is the use of CBNs in sensing implants, which opens up a variety of options for sophisticated diagnosis, real-time monitoring, and therapeutic treatments. Because of their remarkable mechanical qualities, flexibility, and biocompatibility, CBNs—such as graphene and CNTs—make them excellent choices for implanted sensors that track biomechanical parameters within the body.^93^ These sensors offer unparalleled potential to comprehend musculoskeletal dynamics and optimize rehabilitation procedures for injury recovery.^93^ They can give insightful information on joint movements, muscle contractions, and bone stresses.^94^ Moreover, carbon-based nanoparticles are used in implantable devices for biochemical sensing, which allows the identification and measurement of certain biomolecules in the body.^94^

 Implantable sensors that include graphene or CNTs allow for real-time monitoring of metabolites, hormones, and disease biomarkers.^95^ This allows for the early identification of physiological imbalances and the prompt administration of medicinal therapies. CBNs’ optical and magnetic characteristics offer previously unheard-of possibilities for adding imaging capabilities to sensor implants.^96^ Implantable devices have the potential to provide high-resolution imaging of tissues and organs through the utilization of carbon nanomaterials’ distinct optical characteristics.^97^ This can provide valuable information on the course of diseases and physiological processes.^98^ To further improve the diagnostic potential of sensing implants, the magnetic characteristics of these nanoparticles may be used for targeted imaging and monitoring inside certain anatomical areas.^58^ High-fidelity cerebral activity recording and smooth brain-to-machine transmission are two areas in which CBNs have great promise for use in neural sensing and brain-machine interfaces.^99^ Researchers and clinicians can learn more about brain circuits and develop novel treatment strategies for neurological illnesses as well as developments in neuroprosthetics by employing carbon nanomaterials in implanted neural electrodes.^100^ To guarantee the translational potential of these technologies, regulatory and safety concerns must be addressed as the use of CBNs in sensing implants develops.^61^

 Rigorous evaluation of the biocompatibility, long-term stability, and potential immunological responses to CBNs is essential for their safe and effective integration into implantable devices, emphasizing the importance of comprehensive preclinical studies and regulatory oversight.^101^ Thus, the integration of CBNs in sensing implants represents a paradigm shift in biomedical sensing and diagnostics, presenting opportunities for personalized medicine through vaccine development, real-time health monitoring, and transformative interventions in patient care.^61^ The continued exploration of these applications holds immense promise for the future of healthcare and biomedical engineering.^102^

 Scientists find out that for CBNs to be safely and effectively integrated into implantable devices, a thorough assessment of their biocompatibility, long-term stability, and potential immunological responses is necessary.^103^ This emphasizes the significance of thorough preclinical studies and regulatory oversight.^104^ The incorporation of CBNs into sensing implants, therefore, signifies a paradigm change in biomedical sensing and diagnostics and offers prospects for real-time health monitoring, tailored treatment, and revolutionary patient care interventions.^60^ Future research into these applications has enormous potential for the fields of biomedical engineering and healthcare.

###  Antimicrobial applications

 The numerous beneficial characteristics of CBNs present a great deal of promise for the advancement of antimicrobial applications in a range of fields, including environmental remediation and healthcare.^105^ These nanomaterials provide fresh approaches to thwarting drug-resistant organisms, averting infections, and improving cleaning procedures, making them attractive weapons in the battle against microbiological threats. Because of their exceptional antibacterial activity, CBNs—such as graphene and CNTs—are excellent choices for creating antimicrobial surfaces and coatings.^106^ These nanoparticles may be used to generate self-sanitizing materials that efficiently prevent the development and spread of bacteria, viruses, and fungus by adding them to polymers or surface coatings.^107^ These antimicrobial surfaces have a great deal of potential for use in a variety of settings, such as public infrastructure, food packaging, medical equipment, and healthcare facilities.^108^ They can also help reduce the spread of infectious illnesses and maintain public health.^108^ Because of the distinct physicochemical characteristics of CBNs, antimicrobial drugs with improved effectiveness and tailored action may be designed and created.^109^ Through processes including membrane rupture, oxidative stress induction, and interference with microbial adherence, functionalized CNTs and graphene derivatives can display strong antibacterial capabilities.^110^ These antimicrobial agents based on nanomaterials have the potential to be more effective than traditional antimicrobial agents due to their lower potential for resistance development.^111^ They may be applied to the treatment of infectious illnesses, as well as for sterilizing and the creation of new therapeutic modalities.^112^ The prospective uses of CBNs in photothermal and photodynamic treatment for antibacterial purposes have attracted a lot of attention.^113^ By taking advantage of the photothermal characteristics of nanomaterials, such as CNTs and graphene oxide, it is possible to cause localized heating in microbial cells, which results in thermal damage and microbial elimination.^113^

 Furthermore, these nanomaterials’ photosensitizing properties allow for the production of reactive oxygen species in response to light, which facilitates the targeted photodynamic inactivation of pathogens.^114^ Precision antimicrobial treatments with possible answers for localized infections and biofilm elimination are offered by these promising techniques.^115^ Beyond their use in medicine, carbon-based nanoparticles support water treatment and environmental cleanup initiatives by using antibacterial agents.^115^ CNTs and functionalized graphene may be used to create antimicrobial filtration membranes that effectively remove pollutants, pathogens, and microbiological contaminants from water sources.^111,115^ Additionally, the targeted inactivation of microbiological agents in air and water made possible by the introduction of nanoparticles into environmental remediation technologies helps to protect the environment and prevent waterborne illnesses.^116^

 In order to reduce possible dangers, it is critical to address regulatory issues and carry out thorough safety evaluations as the use of CBNs in antimicrobial applications grows.^117^ To guarantee safe deployment and prevent unforeseen consequences, a thorough assessment of the environmental impact, biocompatibility, and long-term toxicity of antimicrobial products based on nanomaterials is required.^118^ Furthermore, the establishment of strong frameworks for the appropriate development and application of these cutting-edge antimicrobial technologies depends on proactive collaboration with regulatory bodies and stakeholders.^119^ As a result, investigating CBNs for antimicrobial applications is essential to tackling the changing problems that microbiological threats in healthcare, environmental sustainability, and public health bring.^120^

 Innovative antimicrobial techniques may be created to battle infectious illnesses, reduce microbial pollution, and expand the paradigm of antimicrobial therapies by utilizing the varied properties of these nanomaterials.^121^ The potential for CBNs to have revolutionary effects on antimicrobial applications is growing as research in this area advances, signaling the beginning of a new age of sophisticated antimicrobial technologies with far-reaching effects on the health and welfare of people everywhere.^122^

###  Treatment and diagnosis

 Recent years have seen the rise of CBNs as potent diagnostic instruments, providing special benefits for the sensitive and targeted identification of pathogens, disease-related compounds, and biomarkers.^123^ These nanomaterials’ remarkable qualities—such as their large surface area, adjustable surface chemistry, and remarkable electrical and optical properties—make them attractive options for next-generation diagnostic platforms.^124^ CBNs, including graphene and CNTs, have been used to create very sensitive biosensors that can identify certain proteins with remarkable accuracy.^125^ Target analytes may be selectively captured and quantitatively measured by functionalizing these nanomaterials with biological recognition components like enzymes, antibodies, or DNA probes.^126^ This opens the door to the possibility of quick and precise point-of-care testing.^127^ These biosensors show promise for a variety of diagnostic uses, such as early illness screening, cancer biomarker profiling, and infectious disease detection, enabling prompt and focused therapies for better patient outcomes.^127^ CBNs can be used as image contrast agents for enhanced diagnostic imaging modalities because of their distinct optical and magnetic characteristics.^128^

 High-contrast viewing of biological structures and disease-related characteristics is made possible by the remarkable imaging capabilities of functionalized carbon nanomaterials, such as carbon dots and graphene quantum dots.^129^ By providing opportunities for enhanced tissue-specific targeting, improved imaging resolution, and multi-modal imaging applications, these nanomaterial-based contrast agents help to advance the development of more precise and insightful diagnostic imaging methods for a wide range of medical conditions.^130^ Novel methods for DNA and RNA sensing have been presented by the use of CBNs in genomic analysis and nucleic acid detection.^131^ The development of nanomaterial-based systems that can capture and amplify nucleic acid sequences opens the door to the sensitive and targeted identification of infectious pathogens, disease-associated genetic markers, and genetic alterations.^132^

 These platforms provide insights into genetic predispositions, disease development, and treatment responses for individualized patient care.^133^ They have enormous promise for use in molecular diagnostics, genomics research, and Tailored drug delivery systems.^134^ The creation of theragnostic platforms—wherein CBNs play dual functions as diagnostic agents and therapeutic carriers—is a result of the confluence of diagnostics and therapies.^134^ Precise diagnosis and targeted therapy administration may be accomplished on a single platform by integrating diagnostic features, such as target-specific identification and imaging, with nanomaterial-based drug delivery systems.^66^ With the ability to simultaneously analyze patients’ medical histories and provide specific therapeutic treatments, these therapeutic techniques have great potential to advance personalized medicine and provide more efficient, individualized treatment plans.^135^

 Comparative studies have shown that both CNTs and graphene exhibit excellent photothermal conversion efficiency, with graphene generally demonstrating higher efficiency due to its higher surface area and better optical absorption properties.^136^ However, CNTs have shown improved selectivity for cancer cells due to their ability to target specific cell surface receptors.^136^ In terms of hyperthermia-based treatments, graphene-based photothermal therapy has been shown to induce significant cancer cell death in vitro and in vivo, while CNT-based therapy has demonstrated promising results in targeted cancer therapy.^137^ Further research is needed to fully elucidate the comparative advantages and limitations of CNTs and graphene in photothermal therapy, but these preliminary findings suggest that both materials hold significant promise for cancer treatment

 In order to ensure the safe and efficient integration of these technologies into clinical practice, it is crucial to address regulatory issues and translational obstacles as the potential for CBNs in diagnostic applications grows.^138^ Securing regulatory clearance and clinical translation of nanomaterial-based diagnostic technologies requires a thorough assessment of their pharmacokinetics, safety profiles, and biocompatibility.^139^ Additionally, in order to create strong frameworks for the ethical development and application of CBNs in diagnostic tools and open the door for their widespread use in healthcare settings, proactive engagement with regulatory bodies, clinical stakeholders, and industry partners is essential.^5^

###  Tissue engineering

 At the forefront of regenerative medicine is tissue engineering, which uses synthetic and biological components to replace or repair damaged tissues and organs.^140^ CBNs have demonstrated great promise and potential in this sector for a variety of applications, providing special qualities that have the ability to completely transform tissue engineering techniques.^141^ Making scaffolds is one of the main uses of CBNs in tissue engineering.^142^ Because of their remarkable mechanical strength, surface area, and conductivity, CNTs, graphene, and carbon nanofibers are excellent choices for building scaffolds that resemble the extracellular matrix of natural tissue.^142^ In the end, these scaffolds can help tissue regeneration by giving cells the structural support they need for adhesion, proliferation, and differentiation.^143^

 To further improve cellular responses and tissue integration, CBNs may be functionalized with bioactive compounds, growth factors, and signaling signals thanks to their variable surface chemistry.^144^ In tissue engineering applications, carbon-based nanoparticles have also shown promise as vehicles for targeted and regulated drug delivery.^145^ Drug-loaded carbon-based carriers can be engineered to offer continuous release of bioactive compounds, growth factors, and therapeutic agents at the site of tissue regeneration by using the large surface area and special physicochemical features of nanomaterials.^146^ By encouraging localized cellular activity and tissue regeneration and reducing off-target effects and systemic exposure, this tailored delivery strategy can maximize the regenerative potential of tissue engineering constructions.^147^ CBNs have important roles in drug delivery and scaffold construction, but they also provide useful tools for bioimaging and tissue growth and development monitoring.^148^ Carbon dots and graphene quantum dots, two examples of nanomaterial-based contrast agents, can be used to visualize and evaluate tissue shape, vascularization, and cellular activity in real time during non-invasive imaging of created tissues.^149^ The capacity to image is essential for assessing the efficacy of tissue regeneration techniques and refining the design of engineered structures to ensure their seamless integration into the host tissue.^150^ CBNs’ remarkable mechanical and electrical conductivity have important ramifications for tissue engineering applications, especially when it comes to synthetic cardiac, neurological, and muscular tissues.^151^ The incorporation of carbon nanomaterials into tissue engineering structures can aid in the creation of mechanically and electrically robust scaffolds, thereby offering a platform for the functional integration and maturation of specialized tissues that are dependent on mechanical coupling and electrical signaling for appropriate operation.^152^ Since CBNs have the potential to improve tissue engineering approaches, it is critical to address translational issues and regulatory concerns in order to guarantee the safe and efficient integration of these materials into clinical practice.^145^

 For regulatory approval and clinical translation, a thorough assessment of the biocompatibility, long-term safety, and tissue-specific effects of constructs using nanomaterials is essential.^5^ Furthermore, in order to create strong frameworks for the ethical development and clinical application of CBNs in tissue engineering and open the door for their revolutionary influence on regenerative medicine and patient care, proactive engagement with regulatory bodies, physicians, and industry partners is imperative.^15^ On Table 2 shows biomedical applications, advantages and limitations of various CBNs.^153-159^

**Table 2:** Biomedical applications, Advantages, & Limitations of various Carbon-based nanomaterials

**Carbon nanomaterials**	**Biomedical applications**	**Advantages**	**Limitations **	**Ref. **
Graphene Graphene **Figure:** 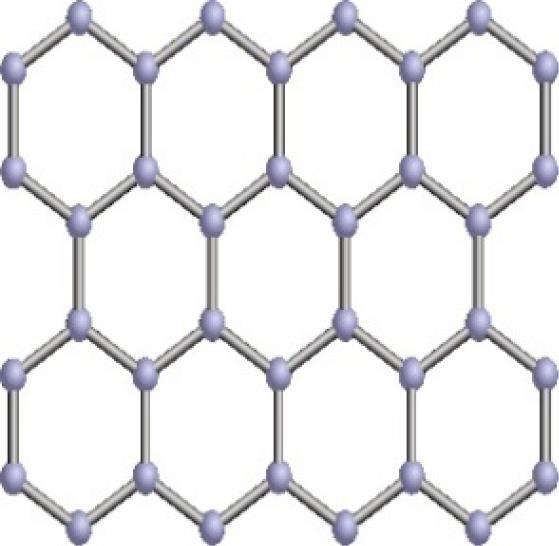	Drug delivery, gene delivery, biosensing, tissue engineering, and bioimaging.	Provide several significant advantages, including extraordinary strength, lightness, flexibility, and great electrical and thermal conductivity. These qualities make it appropriate for a variety of industries, including energy storage, electronics, construction, and healthcare.	Even though graphene is extremely promising, its practical application is limited, mainly because of production issues, expense, and the requirement for changes to improve its functionality. The costly and intricate process of producing high-quality graphene, the requirement to solve its lack of a band gap (which makes it challenging to turn off as an electronic device), and the possibility of toxicity and biocompatibility problems are some of these drawbacks.	^153^
Carbon nanotubes **Figure:** 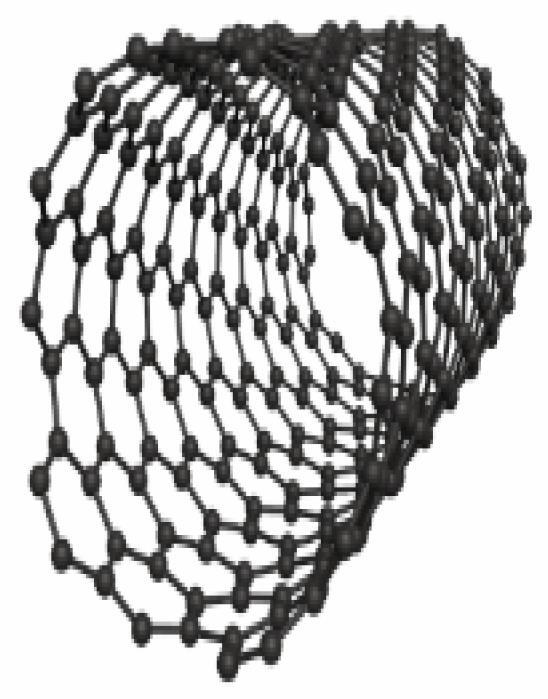	Drug delivery, gene delivery, biosensing, cancer treatment and Imaging technology.	CNTs have various advantages because of their unique characteristics. They are lightweight, robust, and highly conductive, making them ideal for a variety of applications including electronics, composite materials, and biomedical devices. Their large surface area also improves their ability to absorb and transport chemicals, and they are biocompatible, which broadens their application in medical sectors.	Carbon nanotubes (CNTs) have numerous drawbacks, despite their promise features. These include the difficulty of large-scale, cost-effective production, the challenges of managing chirality for consistent semiconducting activity, the possibility of toxicity, and worries about the long-term impacts. Furthermore, present manufacturing methods frequently produce combinations of metallic and semiconducting CNTs, necessitating effective separation procedures for dependable electronics. Scaling up CNTs for practical applications can lessen their extraordinary strength because their qualities are often most noticeable at the tiny level.	^154^
Fullerene **Figure:** 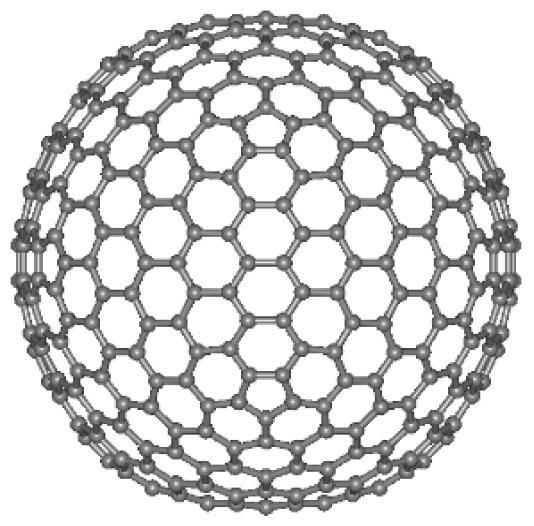	Drug delivery, photodynamic therapy for cancer and antibacterial purposes, biosensors, and tissue engineering.	Fullerenes have multiple advantages due to their unique features, including the ability to act as antioxidants, be good electron acceptors, and have the potential for use in a variety of medical and electronic applications. They are utilized in medicine delivery, cancer treatment, and even cosmetic products, thanks to their capacity to interact with DNA, proteins, and cells. Furthermore, their spherical shape and unique features make them valuable for lubricants and energy storage applications.	Fullerene's use is limited by characteristics such as its inherent insolubility in water, inclination to agglomerate, high cost, and vulnerability to deterioration in light and oxygen. These restrictions limit potential uses in biology and energy storage.	^155,156^
Carbon nano horns **Figure:** 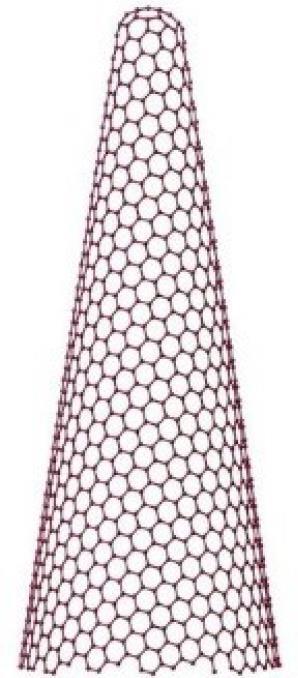	Biosensing, bioimaging, tissue engineering, and cancer therapy are all examples of targeted medication delivery.	Carbon nanohorns (CNHs) have various advantages over other CBNs, such as CNTs. These include high yield, purity, and the capacity to synthesis without using metal catalysts. CNHs also have outstanding features such as a large specific surface area, great chemical stability, strong mechanical strength, and superior conductivity, making them useful in a variety of applications.	CNHs have restrictions resulting from their synthesis and inherent features, including their tendency to congregate and hydrophobicity. These difficulties include functionalization, dispersion, and separation of individual nanohorns. Furthermore, their hydrophobic properties need surface modification for broader uses.	^157^
Carbon quantum dots **Figure:** 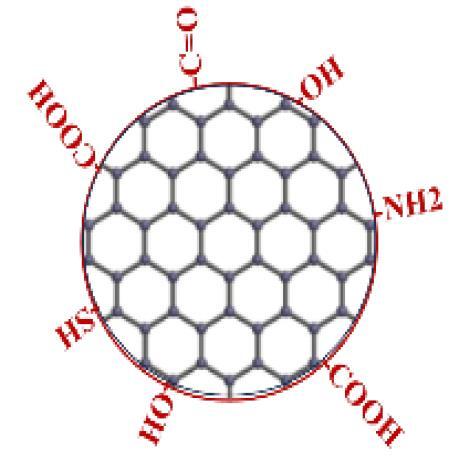	Biomedical imaging, drug delivery, biosensing, and cancer treatment.	Carbon quantum dots (CQDs) have multiple applications due to their unique qualities, which include biocompatibility, low toxicity, tunable fluorescence, and ease of manufacturing. They are a promising nanomaterial for applications such as bioimaging, biosensing, medication delivery, and environmental cleanup.	While CQDs have advantages over standard semiconductor quantum dots, their widespread usage is constrained by a number of issues, including toxicity, low biocompatibility, high cost, and poor chemical inertness. Furthermore, CQDs can display low solubility in aqueous solutions, requiring sophisticated surface modifications for specific applications.	^158^
Carbon onions **Figure:** 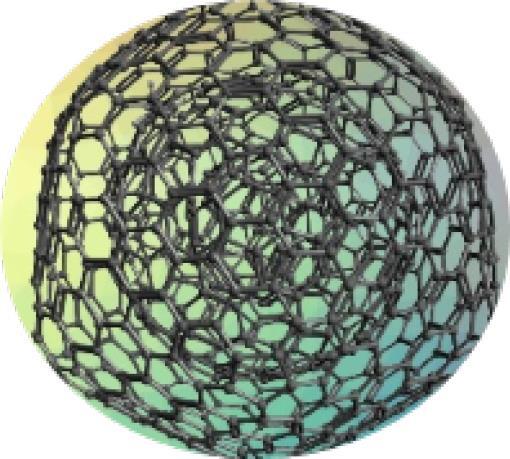	Drug delivery, tissue engineering, bioimaging, and sensing technologies.	Carbon onions, also known as carbon nano-onions (CNOs), provide various benefits due to their distinct structure and features. These include excellent conductivity, mechanical strength, and the ability to store energy. They are also biocompatible and can be employed in different applications, including energy storage and biomedicine.	Carbon onions, while intriguing, have limitations in applicability. They mostly tend to aggregate, lowering their dispersibility and limiting their applicability in a variety of applications. Furthermore, while having high conductivity and vast surface area for applications such as supercapacitors, their energy density and specific capacitance can be relatively low when compared to other materials.	^159^

## Conclusion

 CBN applications in biological medicine, particularly within tissue engineering and diagnostics, have the potential to significantly improve clinical outcomes, regenerative medicine, and personalized medicine. Theragnostic platforms that use CBNs can provide targeted therapeutic treatments and diagnostic evaluation at the same time, resulting in better patient care and healthcare initiatives. Furthermore, CBNs are useful for bioimaging, scaffold construction, drug administration, and improving the mechanical and electrical characteristics of tissue engineering constructions due to their special qualities.

 However, it is crucial to address regulatory issues, carry out thorough assessments of biocompatibility and safety, and actively interact with regulatory bodies and clinical stakeholders to guarantee the safe and successful integration of these technologies into clinical practice. CBN-based biomedicine faces several challenges, including toxicity and biocompatibility, scalability and reproducibility, targeting and delivery, and regulatory frameworks. The long-term effects of CBNs on human health and the environment are not fully understood, and large-scale production remains a significant challenge. Efficient targeting and delivery of CBNs to specific cells or tissues are crucial for effective therapy. Regulatory frameworks are needed to ensure safe and responsible development. Unanswered questions include mechanisms of action, pharmacokinetics and pharmacodynamics, immunogenicity and immune response, and clinical translation. While CBNs have shown promise in various biomedical applications, more research is needed to fully realize their potential and address these challenges.

## Future Perspectives

 CBNs, such as graphene, CNTs, fullerene, and CQDs, are emerging as versatile tools in biomedical research. Their unique physical, chemical, and biological properties enable applications in diagnostics, therapeutics, drug delivery, and tissue engineering. Advancing surface chemistry techniques can help tailor CBNs to specific biological environments, improving biocompatibility and minimizing toxicity. The integration of CBNs with artificial intelligence can revolutionize real-time diagnostics and personalized medicine. The development of multifunctional CBNs for simultaneously targeting disease pathways and delivering therapeutic agents is a promising frontier. Addressing the potential long-term toxicity and environmental impact of CBNs through rigorous evaluation and sustainable synthesis is critical. While promising, the clinical adoption of CBNs remains a challenge due to regulatory hurdles and scalability issues. Collaboration between interdisciplinary teams can expedite the path to clinical applications. Combining CBNs with other nanomaterials, such as metallic nanoparticles or polymeric nanocomposites, may unlock novel biomedical functionalities.

 The review article discusses the limitations of CBNs research, including its scope, bias, methodological variability, lack of standardization, and the lack of understanding of emerging trends. It suggests that further research is needed to understand the mechanisms of action in various biomedical applications, large-scale clinical trials to evaluate safety and efficacy, interdisciplinary collaborations between researchers from diverse disciplines, the development of standardized protocols for synthesis, characterization, and application, and exploring new applications like regenerative medicine, gene therapy, and personalized medicine. The rapidly evolving nature of the field may also limit the capture of emerging trends and future directions. Further research is needed to fully understand the potential of CBNs in biomedicine through standardized toxicity assessments to ensure safety and efficacy. Scalable synthesis methods are being explored to improve production efficiency and reduce costs, while targeted functionalization strategies are being developed to enhance specificity.

## Competing Interests

 The authors of the article declare that they have no financial ties to or connections with any institutions or organization that would present a financial conflict of interest or financial interest in any of the subjects or materials included in it.

## Ethical Approval

 In this review article neither any animals nor any human subjects are involved for research purpose.
